# Risk factors for postoperative throat pain after general anaesthesia with endotracheal intubation at the University of Gondar Teaching Hospital, Northwest Ethiopia, 2014

**DOI:** 10.11604/pamj.2017.27.127.10566

**Published:** 2017-06-16

**Authors:** Biruk Melkamu Gemechu, Endale Gebreegziabher Gebremedhn, Tadesse Belayneh Melkie

**Affiliations:** 1 Department of Anaesthesia, School of Medicine, Gondar College of Medicine and Health Sciences, the University of Gondar, Gondar, Ethiopia

**Keywords:** General anaesthesia, tracheal intubation, postoperative sore throat, risk factors

## Abstract

**Introduction:**

Postoperative sore throat is listed from the top as patients’ most undesirable outcome in the postoperative period. It is believed to originate from mucosal dehydration or edema, tracheal ischemia secondary to the pressure of endotracheal tube cuffs, aggressive oropharyngeal suctioning, and mucosal erosion from friction between delicate tissues and the endotracheal tube. Even if the problem was indicated in many literatures, it has never been studied in our country. The study aimed to assess prevalence and factors associated with postoperative sore throat among patients who were operated under general anesthesia with endotracheal intubation.

**Methods:**

Hospital based cross sectional study was conducted from February 25 - April 10, 2014 in Gondar University hospital. Patient interview and chart review were employed for data collection. Bivariate and multivariate logistic regressions were used to determine the association.

**Results:**

A total of 240 out of 299 patients were included in this study with a response rate of 80.3%. The prevalence of postoperative sore throat within 48 hours after operation was 59.6%. Factors which had association with postoperative sore throat from the multivariate logistic regression were female sex (AOR = 3.3, 95% CI: 1.07, 10.375), repeated number of attempts to intubate (AOR = 3.291, 95% CI: 1.658, 6.531), and the use of nasogastric tube (AOR = 0.41, 95% CI: 0.174, 0.965) respectively.

**Conclusion:**

The prevalence of postoperative sore throat was high in Gondar University Hospital. Awareness creation about the problem should be made for health professionals and postoperative sore throat management protocol need to be introduced.

## Introduction

Regardless of its incidence and some preventive measures, postoperative sore throat (POST) is listed from the top as patients’ most undesirable outcome in the post-operative period. Postoperative sore throat is the commonest patient complaint, significant to patients and frequent postoperative complication [1, 2]. It is well recognized that reporting of sore throat is affected by the method of questioning that is whether the questions asked directly or indirectly [3]. The expression of postoperative sore throat constitutes a number of sign and symptoms. For example sore throat is an ordinary expression of pharyngitis, which by itself can have a number of causes. It may also include a variety of symptoms including pain and discomfort, laryngitis, tracheitis, hoarseness, cough or dysphagia. Postoperatively it seems reasonable that most of the signs and symptoms are the result of mucosal injury which leads to inflammation caused by the process of air way instrumentation. Also its postulated etiology has been associated with mucosal dehydration or edema, tracheal ischemia secondary to the pressure of Endo tracheal tube cuffs, aggressive oropharyngeal suctioning, and mucosal erosion from friction between delicate tissues and the ETT [4, 5].

A number predisposing factors have been identified and the most notable one seems to be the size of the endotracheal tube used, cuff pressure, use of anesthetic spray, female sex, duration of anesthesia, surgical positioning, use of succinylcholine, concurrent use of a nasogastric tube, aggressive oropharyngeal suctioning and the approach to airway management (i.e. ETT, laryngeal mask airway (LMA) or face mask) [6]. The incidence of postoperative sore throat varies in most research studies, but some report the incidence to be less than 15 % and others as high as 64% [7]. The use of a smaller ETT has consistently been shown to significantly reduce the incidence of postoperative sore throat without resulting in problems ventilating patients [8]. Studies have documented the use of 6.5mm - 7 mm ETT for women and 7.5mm - 8 mm ETT for men that resulted in lower rates of postoperative sore throat when compared to larger sized ETT [9]. A study conducted in Nigeria on patients aged 18-77 in 2006 indicated the incidence of POST to be 63%, which was greatly associated with the duration of intubation [10].

Many researchers have suggested that the higher incidence of females reporting POST is more directly related to a tighter fitting ETT than to a distinct difference between the sexes (11). One group of researchers admitted that their choices in ETT sizes did not truly fit the anatomy of patients (they used 8 mm for males and 7.5 mm for females), and suggest that a 7 mm ETT may be a better alternative for females. In fact, several studies seem to advocate a 7 mm sized ETT for female patients and a 7.5 mm ETT for males [4]. The other determinant factor is ETT cuff pressure. There is significant evidence to support that limiting ETT cuff pressures will reduce the incidence of postoperative sore throat [11]. There are some researches that support standard use of manometers, following tracheal intubation the cuff pressure is measured and maintained to a minimum for proper seal and has to be checked intermittently for consistency, but manometers may not be accessible straight away in all institutions [12]. The use of lidocaine lubricants and sprays is controversial; some literatures say it is the persuasive method to decrease POST, others said that it is associated with an increased incidence and severity of POST [13]. William Macewen is honored with the first orotracheal intubation for the purpose of airway protection during anesthesia in 1880 [14]. The incidence of postoperative sore throat varies in most research studies, but some report the incidence to be less than 15% and others as high as 64% [15]. The perioperative and anaesthesia related complications are expected to be higher in the developing countries than the developed countries [16].

Many studies have been performed to determine the incidence of postoperative sore throat and to find measures for its prevention. Most of these studies have been conducted in developed and western populations. The presence of racial difference in the incidence of postoperative complication has been well documented [17–21]. The endotracheal tube has since evolved, but remains a critical tool in the practice of anesthesia in spite of its undesirable effects. There is also another study conducted in United Kingdom showing the incidence to be 63.9% [22]. The prevalence of POST varies from different studies in different countries. In Ethiopia there is no published research found on this topic. A literature search was conducted using the following databases Ethiopian medical journal, journal of health sciences and Ethiopian journal of health and biomedical sciences for articles containing key words in the title or abstract related to postoperative sore throat and ETT. So the incidence of POST is not known in our country. Knowing the incidence of POST and its association factors in our situation helps to recognize the magnitude of the problem and initiates actions to reduce its occurrence. This research can also help as a back ground for future researches on related topic by indicating the incidence rate and associated risk factors of POST in this geographical area. The aim of this study was to assess prevalence and factors associated with postoperative sore throat among patients who were operated under general anaesthesia with endotracheal intubation at the University of Gondar teaching hospital.

## Methods

**Study design, setting and period:** Hospital based quantitative cross sectional study was conducted in the University of Gondar Teaching Hospital main operating theatre recovery room and on the wards from February 25-April 10, 2014.

**Source population:** All patients who were operated at the University of Gondar Teaching Hospital operation theatres.

**Study population:** All adult patients (surgical and obs-gyn patients) who were operated upon under general anaesthesia with endotracheal intubation at the University of Gondar teaching Hospital operation theatres during the study period were included.

**Inclusion criteria:** All adult patients (surgical and obs-gyn patients) who were operated upon under general anaesthesia with endotracheal intubation at the University of Gondar teaching Hospital operation theatres from February 25-April10, 2014 were included.

**Exclusion criteria:** Non cooperative patients, patients with impaired cognitive ability, patients under 18 years of age, unconscious patients after operation, upper respiratory tract infections or throat complications before surgery.

**Dependent variable:** postoperative sore throat (yes/no)

**Independent variables:** Age, sex, endotracheal tube (ETT) size, American Society of Anesthesiologists physical status classification system (ASA), duration of intubation, type of surgery, grade of intubation, intubation attempts, surgical positioning, use of oropharyngeal airway, experience of the anaesthetist, use of nasogastric tube, muscle relaxant choice, cuffed or uncuffed tube.

### Operational definitions

**Postoperative sore throat:** When the patients reported either pain or discomfort or both during swallowing within 48 hours after the operation.

**Sample size and sampling technique:** All consecutive adult patients (surgical and obs-gyn patients) who were operated upon under general anaesthesia with endotracheal intubation at the University of Gondar teaching Hospital operation theatres during the study period were included.

**Data collection procedure:** The data collection procedure included chart review and patient interview using a structured questionnaire. From the chart patient age, ETT size, number of attempts and grade of intubation were recorded. From patient interview using a questionnaire, patients was interviewed 24 hours after the procedure had been done if they are experiencing pain or discomfort during swallowing.

**Data quality control:** To ensure the quality of data pretest of the questionnaire was done at 20 patients at Gondar University Hospital to check for completeness, accuracy and clarity of data obtained from this data collection tool. One BSc holder anaesthetist was involved in data collection and the data collection was supervised by the investigators.

**Data management and analysis:** The data was coded and entered into SPSS version 20 statistical package. All independent variables were analyzed using binary logistic regression with the dependent variable POST and those with a p-value of ≤0.2 form bivariate analysis were fitted to a multivariate logistic regression to check their association with the outcome variable, POST. Odds ratio, 95% confidence interval, and p-value were computed to identify associated factors and to determine the strength of the association. A p-value of <0.05 was considered as statistically significant. Hosmer-Lemeshow test of goodness of fit was performed to check the appropriateness of the model for analysis.

### Ethical considerations

Ethical clearance was obtained from the School of Medicine Ethical Review Committee (Gondar College of Medicine and Health Sciences) before the start of the study. The purposes and the importance of the study were explained & verbal informed consent was obtained from each participant. Confidentiality was maintained at all levels of the study by using anonymous questionnaire and locking the questionnaires securely.

## Results

### Socio-demographic characteristics of the study subjects

A total of 403 elective and emergency patients were operated under anaesthesia during the study period. Of these, 299 patients were operated under general anaesthesia with endotracheal intubation. Out of 299 patients, 43 patients were <18 years old, 9 patients were critically ill and unconscious, 5 patient discharged before 24 hours, and 2 patients refused to participate in the study. A total of 240 patients were included in this study with a response rate of 80.3%. The mean age of patients participated in this study was 34.8 with a standard deviation of 9.9. The minimum and maximum ages were 18 and 71 years respectively. Patients were classified as ASA I, ASA II or ASA III (Table 1).

**Table 1:** Socio-demographic characteristics of the study participants at the University of Gondar Teaching Hospital from February 25 – April 10, 2014 (N=240)

Variable	POST		Total
	No	Yes	
Age (years)			
18-30	50(20.8%)	44(18.3%)	94(39.2%)
31-40	23(9.6%)	63(26.2%)	86 (35.8%)
41-50	17(7.1%)	31(12.9%)	48 (20%)
51-60	6(2.5%)	3(1.2%)	9 (3.8%)
>60	1(0.4%)	2(0.8%)	3(1.2%)
Sex			
Male	52(21.7%)	56(23.3%)	108 (45%)
Female	45(18.8%)	87(36.2%)	132 (55%)
ASA			
ASA I	71(29.6%)	81(33.8%)	152 (63.3%)
ASA II	20(8.3%)	42(17.5%)	62 (25.8)
ASA III	6(2.5%)	20(8.3%)	26 (10.8)
Seniority of the anaesthetist			
Senior	42(17.5%)	81(33.8%)	123 (51.3%)
Junior	55(22.9%)	62(25.8%)	117 (48.8%)

### Factors related with anaesthesia and surgery

Among the various types of surgical procedures, the highest proportion of sore throat occurred in patients who underwent general surgery (58.6% (92/157)). Female patients reported more sore throat than male patients (65.9%vs 51.8% (87/132 vs 56/108)). Multiple intubation attempt appeared to have a direct relationship with the occurrence of postoperative sore throat, one attempt vs multiple intubation attempt (47.5% vs 76.7% (67/141 vs 76/99)). Sore throat was found to be more common with the use of nasogastric tube compared with no nasogastric tube (67.3% vs 57.2% (37/55 vs.106/185)) (Table 2).

**Table 2:** Factors related with anaesthesia and surgery of the study participants in the University of Gondar Teaching Hospital from February 25 – April 10, 2014 (N=240)

Variable	Postoperative sore throat		Frequency: N (%)
	No	Yes	
**Type of operation**			
Elective	47(19.6%)	80(33.3%)	127(53%)
Emergency	50(20.8%)	63(26.3%)	113(47%)
**Type of surgery**			
General	65(27.1%)	92(38%)	157 (65.4%)
Urology	7(2.9%)	4(1.7%)	11 (4.6%)
Orthopedics	1(0.4%)	4(1.7%)	5 (2.1%)
Obs-gyn	24(10%)	43(17.9%)	67 (27.9%)
**Induction agent**			
Ketamine	74(30.8%)	105(43.8)	179 (74.6%)
Propofol	7(2.9%)	20(8.3)	27 (11.3%)
Thiopentone	13(5.4%)	17(7.1)	30 (12.5%)
Ketamine + propofol	3(1.2%)	1(0.4)	4 (1.7%)
**Muscle relaxant**			
Suxamethonium	36(15%)	35(14.6%)	71 (29.6%)
Vecuronium	9(3.8%)	14(5.8%)	23 (9.6%)
Sux + vecuronium	52(21.7%)	94(39.2%)	146 (60.8%)
**Patient position during procedure**			
Supine	96(40%)	136(56.7%)	232 (96.7%)
Lithotomy		3(1.2%)	3 (1.3%)
Prone		2(0.8%)	2 (0.8%)
lateral	1(0.4%)	2(0.8%)	3 (1.3%)
**ETT size**			
6 mm	5(2.1%)	3(1.2%)	8 (3.3%)
6.5 mm	33(13.8%)	64(26.7%)	97 (40.4%)
7 mm	26(10.8%)	25(10.4%)	51 (21.3%)
7.5 mm	33(13.8%)	51(21.2%)	84 (35%)
**Grade of intubation**			
1	81(33.8%)	98(40.8%)	179 (74.6%)
2	16(6.7%)	40(16.7%)	56 (23.3%)
3		5(2.1%)	5 (2.1%)
4			
**Air way use**			
Yes	48(20%)	83(34.6%)	131 (54.6%)
No	49(20.4%)	60(25%)	109 (45.4%)
**Duration of tube in place**			
<=1 hr	19(7.9%)	10(4.2%)	29 (12.1%)
(1-2] hr	43(17.9%)	53(22.1%)	96 (40%)
(2-3] hr	30(12.5%)	69(28.8%)	99 (41.3%)
(3-4] hr	5(2.1%)	6(2.5%)	11 (4.6%)
>4 hr		5(2.1%)	5 (2.1%)

### Prevalence and risk factors for postoperative throat pain

Postoperative sore throat was complained by 143 patients (59.6%). In this study, female sex was significantly associated with POST. Female patients are 3.3 times more likely to develop POST than male patients (AOR = 3.3, 95% CI: 1.07, 10.375). NGT use was also significantly associated with the development of POST. NGT inserted patients were 0.41 times more likely to develop POST than patients who don’t have NGT (AOR = 0.41, 95% CI: 0.174, 0.965). Repeated intubation attempt had also significant association with POST. Patients who had a repeated attempt were 3 times more likely to develop POST than their counter part patients who were intubated during the first attempt (AOR = 3.291, 95% CI: 1.658, 6.531) (Table 3). The variables with a p-value of ≤0.2 from the binary logistic regression that had no association with postoperative sore throat in the multivariate analysis were age, ASA status, ETT size, age, experience of the anaesthetist, muscle relaxant, grade of intubation, and the duration of tube in place.

**Table 3:** Factors associated with postoperative sore throat of patients who underwent general anaesthesia with endotracheal intubation in the University of Gondar Teaching Hospital, February 25 – April 10, 2014 (N=240)

Variables		POST		COR (95% CI)	AOR (95% CI)	P-value
		Yes	No			
Sex	Male	56	52	1.795 (1.066,3.024)	3.3 (1.07,10.375)	0.038
	Female	87	45	1	1	
Nasogastric tube use	yes	37	18	0.653 (0.346,1.231)	0.41 (0.174,0.965)	0.041
	No	109	76	1	1	
Intubation attempt	Single	70	71	3.313(1.862,5.895)	3.291(1.658,6.531)	0.003
	Multiple	76	23	1	1	

**Category reference: COR**= Crude Odds Ratio, **AOR**= Adjusted Odds Ratio **CI**= Confidence Interval

## Discussion

Postoperative sore throat (POST) has been a well-recognized complication after general anaesthesia with endotracheal intubation. While it is generally considered as a minor side effect, POST is important to patients and decreasing its incidence is required to improve anesthetic outcomes [7, 4, 20]. In this study, the prevalence of postoperative sore throat was 59.6%. This finding was high compared with studies conducted in Denmark and Canada [7, 15]. This difference presumably might be due to mucosal ischemia following decreases in blood flow related to high pressures of the tube cuff since there is no standard to the cuff seal point, the erosion and dehydration of delicate mucosal tissues due to dry and nonhumidified gases, aggressive oropharyngeal suctioning and the resulting inflammation in our study. But our finding was low compared with a studies conducted in Nigeria and United Kingdom [10, 22]. This discrepancy could be due to different skills and techniques among anaesthetists.

Female sex had significant association with postoperative sore throat (AOR = 3.3, 95% CI: 1.07, 10.375; p = 0.038). This finding was similar with a study conducted in Burkina Faso (19.29% vs 11.66%; P = 0.01) [20]. This might be due to a gender difference in reporting the adverse outcomes, tighter fitting of the endotracheal tube and there may also be anatomical difference in the larynx between male and female. But there was no association between gender and POST in a study conducted in Nigeria (p = 1.0000, odd ratio = 1.035, 95% CI: 0.5064 - 2.115) [10]. This could be due to they used small sample size.

Number of intubation attempt had significant association with postoperative sore throat (AOR = 3.291, 95% CI: 1.658, 6.531; p = 0.003). Patients who had a repeated attempt were 3 times more likely to develop POST than their counter part patients who were intubated during the first attempt. Our finding was not consistent with studies conducted in Nigeria and Burkina Faso which found no association between number of intubation attempt and postoperative sore throat [10, 20]. This might be due to trauma to mucosa during airway instrumentation and intubation since most of them were carried out by students in our study.

The use of nasogastric tube had strong association with postoperative sore throat (AOR = 0.41, 95% CI: 0.174, 0.965; p = 0.041). This finding was similar with a study conducted in United Kingdom [22]. This could be due to mucosal irritation or damage during insertion. In our study age, ASA, ETT size, experience of the anaesthetist, muscle relaxant, grade of intubation and duration of tube in place had no association with postoperative sore throat. Even though these variables were reported as risk factors for POST in studies conducted in Canada and Pakistan [15, 21]. This might be due to small sample size in our study.

**Limitation of the study:** In this study the sample size was small which was difficult to see whether some variables such as age, ASA status, ETT size, experience of the anaesthetist, muscle relaxant, grade of intubation and duration of tube in place might have an effect on the prevalence of postoperative sore throat in our population. Some important risk factors for POST like tube cuff pressure were not addressed in this study due to lack of the equipment.

## Conclusion

In this study the prevalence of postoperative sore throat was high (59.6%). Female sex, multiple intubation attempts and the use of nasogastric tube were the independent risk factors for postoperative sore throat in Gondar university hospital.

**Recommendations:** Awareness creation should be made about the problem for all health professionals who will be involved in patient management after operation in the University of Gondar hospital. We suggest the introduction of postoperative sore throat management protocols in the University of Gondar hospital. Further study need to be conducted with large sample size to determine whether age, ASA status, ETT size, experience of the anaesthetist, muscle relaxant, grade of intubation and duration of tube in place might have an effect on the prevalence of postoperative sore throat in our population.

### What is known about this topic

- Postoperative sore throat is a common phenomena after general anaesthesia with endotracheal intubation;
- Widely studied in the developed world.

### What this study adds

- Postoperative sore throat is a rarely studied clinical problem in developing nations including Ethiopia;
- Opens a window to develop a prevention strategy as risk factors identified;
- Helps to develop treatment protocols as the common risk factors are identified.

## Competing interests

The authors decalre no competing interest.
